# Inter-rater reliability, sensitivity to change and responsiveness of the orthopaedic Wolf-Motor-Function-Test as functional capacity measure before and after rehabilitation in patients with proximal humeral fractures

**DOI:** 10.1186/s12891-019-2691-0

**Published:** 2019-07-06

**Authors:** Corinna Nerz, Lars Schwickert, Sabine Schölch, Katharina Gordt, Philip-Christian Nolte, Inga Kröger, Peter Augat, Clemens Becker

**Affiliations:** 1 Department for Clinical Gerontology, Robert-Bosch-Hospital, Auerbachstr. 110, 70376 Stuttgart, Germany; 2 Network Aging Research (NAR), Heidelberg University, Heidelberg, Germany; 3 Department of Trauma and Orthopaedic Surgery, BG Trauma Centre Ludwigshafen, Ludwigshafen, Germany; 4 Institute for Biomechanics, BG Unfallklinik Murnau, Murnau, Germany; 5 Institute for Biomechanics, Paracelsus Medical University, Salzburg, Austria

**Keywords:** Wolf motor function test, Reliability, Sensitivity to change, Responsiveness, Orthopaedic assessment, Shoulder function, Proximal humeral fracture

## Abstract

**Background:**

The incidence of proximal humeral fractures (PHF) increased by more than 30% over the last decade, which is accompanied by an increased number of operations. However, the evidence on operative vs. non-operative treatment and post-operative treatments is limited and mostly based on expert opinion. It is mandatory to objectively assess functional capacity to compare different treatments. Clinical tools should be valid, reliable and sensitive to change assessing functional capacity after PHFs. This study aimed to analyse inter-rater reliability of the videotaped Wolf-Motor-Function-Test-Orthopaedic (WMFT-O) and the association between the clinical WMFT-O and the Disability of the Arm, Shoulder and Hand (DASH) and to determine the sensitivity to change of the WMFT-O and the DASH to measure functional capacity before and after rehabilitation in PHF patients.

**Methods:**

Fifty-six patients (61.7 ± 14.7 years) after surgical treatment of PHF were assessed using the WMFT-O at two different time points. To determine inter-rater reliability, the videotaped WMFT-O was evaluated through three blinded raters. Inter-rater agreement was determined by Fleiss’ Kappa statistics. Pearson correlation coefficients were calculated to assess the association between the clinical WMFT-O and the video rating as well as the DASH. Sensitivity to change and responsiveness were analysed for the WMFT-O and the DASH in a subsample of forty patients (53.8 ± 1.4 years) who were assessed before and after a three week robotic-assisted training intervention.

**Results:**

Inter-rater agreement was indicated by Fleiss’ Kappa values ranging from 0.33–0.66 for functional capacity and from 0.27–0.54 for quality of movement. The correlation between the clinical WMFT-O and the video rating was higher than 0.77. The correlation between the clinical WMFT-O and the DASH was weak.

Sensitivity to change was high for the WMFT-O and the DASH and responsiveness was given. In comparison to the DASH, the sensitivity to change of the WMFT-O was higher.

**Conclusion:**

The overall results indicate that the WMFT-O is a reliable, sensitive and responsive instrument to measure more objectively functional change over time in rehabilitation after PHF. Furthermore, it has been shown that video assessment is eligible for studies to ensure a full blinding of raters.

**Trial registration:**

Clinicaltrials.gov, NCT03100201. Registered on 28 March 2017. The trial was retrospectively registered.

**Electronic supplementary material:**

The online version of this article (10.1186/s12891-019-2691-0) contains supplementary material, which is available to authorized users.

## Background

The individual and societal burden of musculoskeletal injuries, in particular of bone fractures remains to be underestimated. Proximal humeral fractures (PHF) are among the leading causes of functional impairment in patients after trauma resulting in limitations in basic, instrumental and advanced activities of daily living. PHFs and wrist fractures are recognized as the most common fractures of the upper extremities accounting for more than 20% of hospital admissions caused by a fracture [1]. In patients over 40 years of age, the proportion of PHFs increases to 76% [2]. Since 2000 in Germany the incidence of PHFs has risen from 178 to 246/100.000 inhabitants/year [3]. In addition, an analysis from Bauer and colleagues showed that one third of the patients are still integrated into the work process [4]. Due to the demographic change, a further increase in the number of PHFs is expected [5, 6]. This will lead to a significant increase in PHFs requiring operative or non-operative treatment and post-trauma hospitalization and rehabilitation.

To date, there is no robust evidence-based consensus on rehabilitation after PHF regarding standardisation of content, duration, intensity or frequency [7–9]. One essential requirement to perform controlled studies on surgical and rehabilitation interventions is the availability of objective, reliable and valid assessments. If possible, these assessment tools should be blinded to treatment allocation. To assess functional capacity and task performance after PHF, at least two types of measures are required: patient-reported outcomes using questionnaires to assess activities of daily living and a supervised clinical-based assessment to measure functional capacity of the patients [10, 11]. The Disability of the Arm, Shoulder and Hand (DASH) questionnaire is the most commonly used questionnaire for assessing activities of daily living after shoulder and arm injuries [10]. A clinically administered assessment of the functional capacity including the quality of movement of PHF patients is the Wolf-Motor-Function-Test-Orthopaedic (WMFT-O) which has previously been assessed regarding re-test reliability, inter-rater reliability, and internal consistency [12]. One further property required of an outcome measurement is the sensitivity to change. It is understood as the ability to describe changes occurring during a treatment or observational period. The particular meaning of this property is described by the fact that positive changes in a given period represent the classic therapeutic goal [13]. Beyond assessing functional change in clinical state over time with sufficient sensitivity to change [14–16] clinical-based assessment tools also need a high responsiveness to decide if a change over time is clinically meaningful [17, 18]. There are no studies that examined the sensitivity to change of the WMFT or the WMFT-O which shows the need to consider also the change over time of the functional capacity and the quality of movement of the WMFT-O.

The aims of this study were 1st to test the inter-rater reliability of the videotaped WMFT-O, 2nd to describe the correlation of the functional capacity assessed by the WMFT-O and the activities of daily living from a patient perspective assessed by the DASH questionnaire, and 3rd to describe the sensitivity to change and the responsiveness of the WMFT-O and the DASH in a group of patients with PHFs.

## Methods

### Patients

For testing inter-rater reliability of the videotaped WMFT-O two patient populations were assessed. The first sample was a group of sixteen patients with an age range from 75 to 90 years with surgical treatment after PHF [12]. Due to the funding guidelines of the sponsor of the study (“Deutsche Gesetzliche Unfallversicherung (DGUV)”) only persons up to 69 could be included into the intervention study. This decision is based on the study approach to be able to quickly re-integrate patients after PHF into everyday work [19]. The second group consisted of 40 patients with an age range from 34 to 69 years with surgical treatment after PHF participating in a randomised controlled trail to measure the effectiveness of robot-assisted training added to conventional rehabilitation [19]. The proximal humeral fractures of both patient groups were surgically fixed by plate osteosynthesis, screw fixation, endoprostheses or humeral nails. The second patient group was recruited at three different clinical sites in Germany and patients were randomised into an intervention group and a control group. They were assessed before randomisation (baseline) and after completing an intervention period of 3 weeks (reassessment). At baseline, cognition was assessed by the Short Orientation-Memory-Concentration Test [20] as well as visual acuity, gait speed (10-m walk [21]), level of pain in the affected arm, ability to work, disability of the arm, shoulder and hand (DASH [22]), range of motion of the affected arm (goniometer measurement [23]), and motor function of the affected arm and shoulder (WMFT-O; [12]). Clinical reassessment directly after the intervention assessed disability of the arm, shoulder and hand (DASH) as well as range of motion and functional capacity (WMFT-O). The WMFT-O was videotaped at both time points. To analyse the sensitivity to change and the responsiveness of the WMFT-O the second subsample with 40 patients was analysed. Both studies were approved by the ethical committee of the University of Tübingen and are in agreement with the Declaration of Helsinki. All participants gave written informed consent.

### Outcome measures

The **WMFT-O** is an adapted version of the basic WMFT [24] with good clinical inter- and intra-rater reliability. The modified version for shoulder injuries was developed by our group [12]. The WMFT was developed to measure functional improvement between the beginning and the end of therapy. The WMFT-O is supervised and includes 20 arm motion tasks used in daily living that are evaluated in terms of functional capacity and quality of movement. The single items are progressing from coarse movements in the elbow and shoulder area to more complex and dexterous tasks in the fingers and the hand area. The endpoint is a total score calculated from the ratings of the functional capacity and the quality of movement (5 is the best value, 0 the worst). The total WMFT-O score ranges from 0 to 100 points with lower scores indicating greater disability (Additional file 1: Video tutorial Rating Scale of the WMFT-O: https://youtu.be/WQUSl2XQJMY and Additional file 2: Video tutorial performance instructions of the WMFT-O: https://youtu.be/K6g3Z_ibNa8). First a clinical rating was performed by pre-trained assessors. As a next step each videotaped WMFT-O (baseline and reassessment, *n* = 112) was assessed by three out of five pre-trained raters (4 physiotherapists, 1 occupational therapist).

The first module of the **DASH questionnaire** was used to measure self-perceived activity and limitation of shoulder, arm and hand function [22]. The DASH is considered as a valid and reliable non-supervised test [25–28]. The questionnaire consist of 30 questions to assess the restrictions related to the function and activity of the shoulder, arm and hand in daily living, as well as self-esteem and potentially existing symptoms of the shoulder, arm and hand, such as pain or prickle. The endpoint is a total score calculated from the ratings of the individual responses (1 is the best value, 5 the worst). The total DASH score ranges from 0 to 100 points with higher scores indicating greater disability.

### Data analysis

All statistics and outcome analyses were performed using RStudio software (Version 1.1.383). The inter-rater agreement of the video rating was determined by Fleiss’ Kappa statistics with corresponding confidence intervals (CI). Pearson correlation coefficients were calculated to assess the association between the WMFT-O clinical rating and the video rating as well as between the WMFT-O clinical rating and the DASH questionnaire.

*Sensitivity to change* was defined as the ability of an instrument to respond to changes in the measured construct, regardless of whether the change is relevant or meaningful to the decider [14, 18]. In order to analyse the sensitivity to change of the WMFT-O, the change scores and the standardized effect size as well as the standardized response mean [29] were calculated. Change scores imply the delta of the results between the baseline- and reassessment. This score represents the extent of which a patient changes in performance in the corresponding test. The Wilcoxon test was calculated to compare the baseline and the reassessment score (*p* > 0.01). The standardized effect size was calculated by dividing the change score by the standard deviation of the baseline score [30]. The standardized response mean was calculated by dividing the change score by the standard deviation of that change score [31]. Values < 0.01 for the standardized effect size are considered as very small, < 0.2 as small, < 0.5 as medium, < 0.8 as large, < 1.2 as very large and < 2.0 as huge [32]. Husted and colleagues [29] interpreted the values of the standardized effect size and the standardized response mean considering the same benchmarks (< 0.2 for trivial, 0.2 to< 0.5 for small, 0.5 to< 0.8 for moderate, and 0.8 or greater for large). Cohen’s threshold values are > 0.8 equates to large, > 0.5 to medium and > 0.2 to small effect sizes and intended for intervention studies, but are sometimes used to apply sensitivity to change of questionnaires [31].

*Responsiveness* was defined as the ability of an instrument to measure a meaningful or important change in a clinical state [14, 18] and is usually reported through the minimal important difference [33, 34]. For being considered as important, a change score of a measure should equal or exceed its minimal important difference estimate. Minimal important difference values were calculated using two commonly used effect size estimates in the literature: 0.3* standard deviation of the baseline score and 0.5* standard deviation of the baseline score [34, 35].

## Results

The study sample consisted of two groups. The first subsample included 16 older patients with a mean age of 81.4 years (range 75–90 years). The second subsample includes 40 younger patients with a mean age of 53.8 years (range 39–69 years). The subject characteristics are listed in Table 1.

**Table 1:** Participant demographics

Characteristic	1st Subsample	2nd Subsample	Total sample
Subjects (n)	16	40	56
Gender			
Female (%)	13 (81.2%)	25 (62,5%)	38 (67.9%)
Age (years)			
Mean (SD)	81.4 (1.1)	53.8 (1.4)	61.7 (14.7)
Minimum	75	39	39
Maximum	90	69	90
Affected Arm			
Right (%)	10 (62.5%)	17 (42.5%)	27 (48.2%)
Arm Dominance ^a^			
Left (%)	1 (6.3%)	5 (12.5%)	6 (10.9%)
Right (%)	15 (93.7)	34 (85.0%)	49 (89.1%)
Dominant arm affected (%)	9 (56.3%)	17 (42.5%)	26 (47.3%)

*n* Number, *SD* Standard deviation; ^a^Dominant arm could not be determined in one subject.

### Inter-rater reliability of the videotaped WMFT-O

Table 2 lists inter-rater reliability across all items of the videotaped WMFT-O. The Fleiss’ Kappa values ranged between 0.35 and 0.67 (CI = 0.27 to 0.73) for the functional capacity and between 0.27 and 0.54 (CI = 0.21 to 0.60) for the quality of movement. Grip strength (task 15) was not considered since the task could not be analysed from the video recordings.

**Table 2:** Inter-rater reliability across all items of the video WMFT-O; *n* = 56 (*p* < 0.005)

Task	Functional Capacity	Quality of Movement
	Inter-rater reliability Fleiss’ Kappa (95% CI)	Inter-rater reliability Fleiss’ Kappa (95% CI)
1. Forearm to table lateral	0.41 (0.35–0.48)	0.30 (0.24–0.37)
2a/b. Forearm to box 15/30 cm lateral	0.47 (0.41–0.53)	0.39 (0.34–0.44)
3. Forearm to box lateral with weight	0.64 (0.57–0.71)	0.54 (0.48–0.60)
4. Extend elbow lateral	0.49 (0.44–0-55)	0.46 (0.41–0.51)
5. Extend elbow lateral with weight	0.56 (0.50–0.62)	0.43 (0.38–0.49)
6. Hand to table frontal	0.60 (0.53–0.67)	0.38 (0.32–0.45)
7a/b. Hand to box 15/30 cm frontal	0.55 (0.49–0.61)	0.51 (0.46–0.57)
8. Hand to box frontal with weight	0.67 (0.60–0.73)	0.53 (0.48–0.59)
9. Reach and retrieve frontal	0.49 (0.43–0.55)	0.44 (0.37–0.50)
10. Lift can frontal	0.59 (0.52–0.65)	0.50 (0.44–0.56)
11. Lift pencil frontal	0.60 (0.53–0.67)	0.45 (0.38–0.51)
12. Lift paper clip frontal	0.51 (0.45–0.58)	0.39 (0.32–0.45)
13. Stack checkers frontal	0.50 (0.44–0.57)	0.34 (0.27–0.40)
14. Flip cards frontal^a^	0.49 (0.43–0.56)	0.37 (0.30–0-43)
16. Turn key in lock frontal	0.35 (0.27–0.42)	0.27 (0.21–0.33)
17. Fold towel frontal	0.49 (0.42–0.55)	0.42 (0.36–0.47)
18. Lift basket frontal	0.47 (0.41–0.53)	0.43 (0.37–0.48)

*CI* Confidence Interval, ^a^ task 15 (grip strength) was omitted.

### Correlation between the WMFT-O clinical and video rating and the DASH

**Fig. 1:**
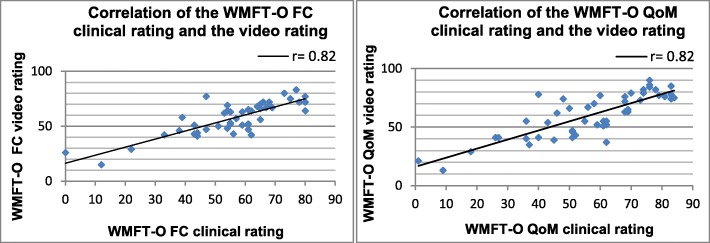
Correlation of the WMFT-O functional capacity and quality of movement clinical rating and video rating (**FC** Functional capacity, **QoM** Quality of movement; Pearson correlation coefficient (r); *p* < 0.001)

Pearson correlation coefficients (r) of the WMFT-O clinical rating and the video rating were high for all three raters and highly significant (functional capacity and quality of movement r≥ 0.82, *p* < 0.001) (Fig. 1). After reducing missing or occluded video recordings, the Pearson correlation coefficient for the functional capacity could be calculated for 49 subjects and for 48 subjects for the quality of movement. Due to the uniform results of the three different raters, in Fig. 1 only the ratings of the first rater are shown.

**Fig. 2:**
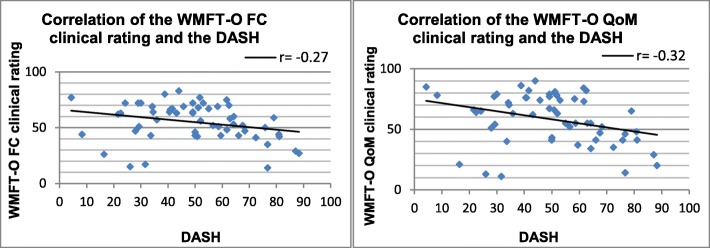
Correlation of the WMFT-O functional capacity and quality of movement clinical rating and DASH (**FC** Functional capacity, **QoM** Quality of movement; Pearson correlation coefficient (r); *p* < 0.05)

The correlation between the WMFT-O clinical rating and the DASH questionnaire was significantly weak at baseline (functional capacity r = − 0.27 and quality of movement r = − 0.32, *p* < 0.05) (Fig. 2). The Pearson correlation coefficient for the DASH could be calculated for 56 subjects.

### Sensitivity to change and responsiveness

The sensitivity to change and the responsiveness indices of the WMFT-O and DASH are listed in Table 3. For the WMFT-O the clinical rating was carried out locally in the individual study centres by one trained rater and the DASH was completed by the subject himself on site. The baseline and the reassessment scores for the WMFT-O functional capacity and quality of movement as well as the DASH differ significantly from each other (*p* < 0.01). The WMFT-O and the DASH demonstrated large standardized effect sizes, ranging between 0.8 and 0.9 (Table 3). Large standardized response means were also obtained for the WMFT-O functional capacity, quality of movement and the DASH. The standardized response mean for the DASH was slightly less sensitive to change when compared to the WMFT-O functional capacity and quality of movement. The minimal important differences for the WMFT-O functional capacity ranged between 5.0 (0.3* standard deviation of the baseline score) and 8.3 (0.5* standard deviation of the baseline score) and for the WMFT-O quality of movement between 6.3 (0.3* standard deviation of the baseline score) and 10.5 (0.5* standard deviation of the baseline score) and are comparable to the minimal important differences of the DASH (Table 3).

**Table 3:** Sensitivity to change and responsiveness of the clinical WMFT-O and DASH; *n* = 40

Parameter	Baseline Mean (SD)	Reassessment Mean (SD)	CS (SD)	SES	SRM	MID (0.3SD_b_)	MID (0.5SD_b_)
WMFT-O							
FC	54.3 (16.5)	69.2 (12.8)	14.9 (10.1)	0.9	1.5	5.0	8.3
QOM	57.7 (20.9)	76.1 (14.8)	18.4 (14.1)	0.9	1.3	6.3	10.5
DASH	51.9 (20.0)	36.7 (16.6)	−15.2 (12.6)	0.8	1.2	6.0	10.0

*SD* Standard deviation, *CS* Change score, *SES* Standardized effect size, *SRM* Standardized response mean, *MID* Minimally important difference, *SD*_*b*_ Standard deviation Baseline, *WMFT-O* Wolf-Motor-Function-Test-Orthopaedic, *FC* Functional capacity, *QoM* Quality of Movement.

## Discussion

Our study on patients with fractures of the proximal humerus demonstrated high sensitivity to change and good responsiveness of the orthopaedic Wolf-Motor-Function-Test indicating its usefulness as a functional capacity assessment tool in operative and rehabilitation studies.

This study also found moderate inter-rater reliability for the videotaped version of the WMFT-O (Table 2). According to Landis and Koch [36] the calculated Fleiss’ Кappa values for the functional capacity and quality of movement can be interpreted as a fair to substantial agreement. The observed agreements were weaker than in a previous clinical study using a non-videotaped assessment of WMFT-O in similar patients [12]. Another publication investigating the reliability of the neurological based WMFT-N version [37] also had higher interrater agreement. The lower agreement of the videotaped measurement could have several reasons. Possibly the training session of the video raters were not enough. A further explanation for the lower agreement could also be an inadequate positioning of the chosen video camera and a different camera position might lead to better results. The performance of the tasks on such videos (e.g. in task 16- “Turn key in lock frontal”, Fleiss’ Kappa for functional capacity =0.33 and quality of movement =0.27; Table 2) was not easy to identify which resulted in more diverse ratings between the raters. Moreover, an inadequate description of the correct end-position in task 1 (“Forearm to table lateral”, Fleiss’ Kappa functional for capacity =0.41 and quality of movement =0.30; Table 2) lead to different opinions about fulfilling or not fulfilling the task. A possible solution could be reducing the prescribed tasks to improve rater agreement. The tasks that were poorly rated could potentially be omitted and a future short version of the WMFT-O could be provided. According to Landis and Koch [36], an inter-rater reliability of 0.01 to 0.20 is considered as a slight agreement and 0.21 to 0.40 as a fair agreement. If these values were used as a basis to decide which items of the WMFT-O could be deleted in a short version, this would delete task 1 and 16 due to the low inter-rater reliability of the functional capacity and quality of movement as well as task 2ab, 12, 13 and 14 due to the low inter-rater reliability of the quality of movement (Table 2).

A third aspect is the calculation of different statistical measures. Inter-rater agreement for two different raters, as calculated in Oberle and colleagues 2018 [12] should be determined by weighted Cohen’s Kappa (Кw) statistics [38]. For three or more raters, Fleiss’ Kappas is recommended as the method of choice. The Fleiss’ Kappa assumes that the examiners were randomly selected from a group of available examiners. Cohen’s Kappa, on the other hand, assumes that examiners have been specifically selected and trained. Therefore, the probability of agreement in the Fleiss’ Kappa and the Cohen’s Kappa is estimated in different ways. In some cases, Fleiss’ Kappa in general may produce lower values even if the agreement is actually high as described before [39]. That in turn may be a possible explanation why the inter-rater reliability values of the video-based WMFT-O calculated by Fleiss’ Kappa (Table 2) were lower than the inter-rater reliability values of the clinically WMFT-O as reported by Oberle and colleagues [12] and calculated by Cohen’s Kappa.

We found a very strong correlation between the WMFT-O clinical baseline rating and the video baseline ratings for the functional capacity and a strong correlation for the quality of movement according to the standards of Evans [40].

To assess treatment effects of interventions for musculoskeletal conditions, functional capacity and personal activity need to be evaluated. Therefore, the WMFT-O has to be augmented by other methods to evaluate levels of disability, activity and participation. A widely applied method is the DASH. It is expected that the correlation between activity levels and functional capacity is often less than anticipated. The correlation between the clinical-based measures (WMFT-O clinical rating of the functional capacity and the quality of movement) and the patient-reported questionnaires (DASH) at baseline was indeed weak [40]. One aspect is that patients do not return to their activity levels due to psychological problems such as insufficient self-efficacy. Other aspects are methodological problems. The DASH is not explicitly designed for the affected arm. This means that in instances where tasks are mostly carried out with the dominant hand (e.g. turning a key in a lock) the restriction in the non-dominant arm, shoulder and hand are not necessarily being captured through the DASH. This is only the case if the affected hand is also the dominant hand. This misjudgement could be avoided if care is taken that when answering the questions of the DASH, the assessment of the restriction always relates to the performance of the activity with the affected shoulder, arm or hand. If it is not possible to carry out the activity of daily living with the affected shoulder, arm or hand the patient must be able to imagine the execution of the activity of daily living and the possible restrictions as best as possible and then answer the question.

One study from Wu and colleagues [41] developed the streamlined WMFT which includes the performance rating of 6 timed tasks for neurological patients. They found a low effect size for the streamlined WMFT and the original WMFT [41]. In comparison to this study the WMFT-O had a large effect size for both the functional capacity and the quality of movement and can thus be regarded as being sensitive to change over time according to the published standards [29, 32]. This result was confirmed by the absolute values of the standardized response mean, which can be considered higher than the standardized effect sizes for both the functional capacity and the quality of movement. This is a relevant finding in terms of clinical use to provide a more objective and sensitive measure for assessing functional capacity and quality of movement of the upper extremities for patients in orthopaedic rehabilitation and may improve current assessments which currently are mostly subjective. Compared to the WMFT-O, the sensitivity to change of the DASH was lower. These findings indicate that the WMFT-O is a more sensitive outcome measure for assessing functional change over time through rehabilitation in patients with PHF. MacDermid and colleagues [42] and Westhphal and colleagues [11] determined the sensitivity to change of the DASH for patients after wrist fractures. They found higher values for the effect size of the DASH in patients with wrist fractures after 0–3 months. After 3–12 months they observed to be somewhat lower [11]. This indicates that the standardized effect sizes and standardized response mean depend on the point of time of the baseline- and reassessment. In the first 12 weeks after baseline, a large treatment effect can be expected. The next three quarters might lead to further improvements but the effect sizes will be smaller. In our study, the baseline assessment was conducted approximately one month after surgery. In order to find out whether the WMFT-O can also be used to assess long-term therapy results, a future study should be carried out including longer therapy intervals assessing upper extremity functional capacity measured with the WMFT-O after three month. A similar relation was found for the DASH questionnaire. In conclusion, both the WMFT-O and the DASH are responsive and complimentary assessment instruments in order to measure the functional change in patients with PHF.

## Conclusion

The WMFT-O is a responsive instrument to objectively measure patient functional change in younger and older patients with PHF. It showed a somewhat higher sensitivity to change in younger and older patients with PHF compared to the DASH. For assessing treatment success of interventions and rehabilitation functional capacity could be measured by the WMFT-O but it should be augmented by another method (for example the DASH) to enable the evaluation of levels of disability, activity and participation. We recommend minor modifications of the camera positions and of the descriptions of some movement tasks for the videotaped WMFT-O. After a detailed training of the rater and taking into account these changes we also recommend the application of a video based assessment as this would allow robust blinding of assessors which often is not the case due to contamination of therapists and assessment staff. Furthermore a short version of the WMFT-O would be desirable.

## Additional files

Additional file 1: Video tutorial Rating Scale of the WMFT-O. Video tutorial with detailed instructions how to rate the single items of the WMFT-O (functional capacity and quality of movement) in English. (MP4 6957 kb)

Additional file 2: Video tutorial performance instructions of the WMFT-O. Video tutorial with detailed instructions how to perform the single items of the WMFT-O in English. (MP4 21016 kb)

## Data Availability

The datasets used and/or analysed during the current study are available from the corresponding author on reasonable request.

## Abbreviations

CI: Confidence Intervals
CS: Change Score
DASH: Disability of the Arm, Shoulder and Hand
FC: Functional Capacity
MID: Minimally Important Difference
PHF: Proximal Humeral Fractures
QoM: Quality of Movement
r: Pearson correlation coefficients
SD: Standard Deviation
SD_b_: Standard Deviation baseline
SES: Standardized Effect Size
SRM: Standardized Response Mean
WMFT-O: Wolf-Motor-Function-Test-Orthopaedic
Кw: Cohen’s Kappa
